# Link between Insulin Resistance and Obesity—From Diagnosis to Treatment

**DOI:** 10.3390/diagnostics12071681

**Published:** 2022-07-10

**Authors:** Jakub Gołacki, Małgorzata Matuszek, Beata Matyjaszek-Matuszek

**Affiliations:** 1 Department of Endocrinology, Diabetology and Metabolic Diseases, Medical University of Lublin, Jaczewskiego 8, 20-954 Lublin, Poland; beata.matyjaszek-matuszek@umlub.pl; 2 Student’s Scientific Society at the Department of Endocrinology, Diabetology and Metabolic Diseases, Medical University of Lublin, 20-954 Lublin, Poland; matuszek.ma@wp.pl

**Keywords:** insulin resistance, obesity, pathogenesis, causes, diagnosis, treatment

## Abstract

Insulin resistance (IR) has become a common health issue in medical practice. There are no detailed data on IR prevalence, but it is an increasing problem due to its close association with obesity. However, IR is not considered as a separate nosological entity and the diagnostic criteria are not well defined, which leads to overdiagnosis of IR and an inappropriate approach. This review aims to summarize the available literature on IR pathophysiology, its relationship with obesity, as well as diagnostic methods, clinical presentation and treatment. Excessive energy intake results in cell overload that triggers mechanisms to protect cells from further energy accumulation by reducing insulin sensitivity. Additionally, hypertrophied adipocytes and macrophage infiltration causes local inflammation that may result in general inflammation that induces IR. The clinical picture varies from skin lesions (e.g., acanthosis nigricans) to metabolic disorders such as diabetes mellitus or metabolic-associated fatty liver disease. There are numerous IR laboratory markers with varying sensitivities and specificities. Nutrition changes and regular physical activity are crucial for IR management because a reduction in adipose tissue may reverse the inflammatory state and consequently reduce the severity of insulin resistance. In cases of obesity, anti-obesity medications can be used.

## 1. Introduction

The diagnosis of insulin resistance is a common reason for patients (usually females) to visit an endocrinology clinic, diabetes clinic, family doctor or even a dietary clinic. Since the introduction of this term in 1931 by Wilhelm Falta, a Bohemian physician working in Vienna, the understanding of this phenomenon and the methods for laboratory diagnosis have significantly developed (Rosalyn Yalow and Solomon Berson were awarded the Nobel Prize in 1977 for the development of a sensitive method for detecting insulin in the blood), but there are still no clear guidelines for its diagnosis, management and treatment [1]. Given the above, we have prepared a collective summary of up-to-date information on the pathogenesis, clinical picture, laboratory diagnostics and treatment of insulin resistance.

Insulin resistance (IR) is a state of decreased cellular sensitivity to insulin despite an elevated or normal serum insulin concentration, which is an expression of their dysfunction. The reduction in insulin utilization prompts pancreatic beta cells to produce and release increasing amounts of insulin in order to break down cellular resistance and correct the relative insulin deficiency. This developing hyperinsulinemia is an effective compensatory mechanism that allows for insulin action in mild to moderate IR. Such a defect in insulin signaling not only impairs the utilization of glucose and lipids, but also causes accelerated atherosclerosis and the development of organ complications such as hypertension, atherogenic dyslipidemia, metabolic associated fatty liver disease, and many others [2]. However, IR is not recognized as a separate nosological entity, but merely as a pathogenic phenomenon of many metabolic diseases. In view of the latest reports, IR can be treated as a synonym for overweight and obesity due to their mutual pathogenetic relationships. Therefore, our analysis is based mainly on these two pathologies.

## 2. Pathophysiology of IR Development

IR can have many causes, as shown in Table 1, but by far the most common, especially in developed countries, is during the course of obesity [3,4]. In view of the growing number of people with obesity in the world and the constant threat of its numerous complications, we have observed increasing interest in studies that explore the essence of the pathogenesis of IR.

### 2.1. Physiological Action of Insulin

**Figure 1:**
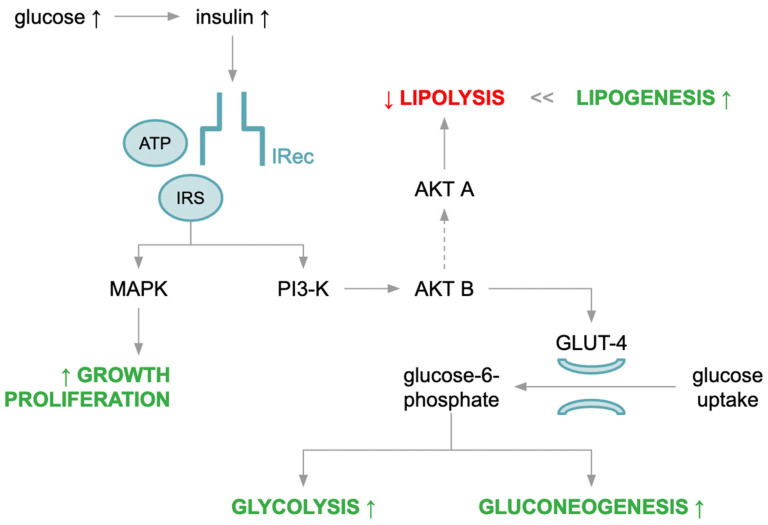
Insulin action physiology.

Insulin is a protein hormone released into the blood by endocrine pancreatic beta cells in response to a glucose stimulus during a meal. It is believed to have pleiotropic effects depending on the effector, and is an anabolic hormone responsible for the production of compounds composed of simple substrates, especially in adipocytes, hepatocytes and myocytes. Insulin exerts its metabolic action through a specific membrane receptor. This glycoprotein with tyrosine kinase properties is composed of two alpha-subunits and two membrane-spanning beta-subunits (transmembrane and intracellular parts) linked by disulfide bonds. After insulin binds to its receptor, a cascade of interrelated reactions involving secondary transmitters is activated leading to the specific metabolic effects shown in Figure 1.

Initially, there is a change in receptor configuration, activating autophosphorylation of the beta subunit, and thanks to ATP, the next step involves phosphorylation of tyrosine residues of intracellular substrate proteins (insulin receptor substrate, IRS), the most important of which are IRS-1 and IRS-2, among others (Shc adaptor protein, Gab-1 and Cbl) [5]. Activated IRSs are able to phosphorylate the substrates of two important intracellular signaling pathways related to phosphatidylinositol 3-kinase (PI-3K) and mitogen-activated protein kinase (MAPK). PI-3K is responsible for metabolic activity due to its major role in insulin-stimulated intracellular translocation of glucose transporter type 4 (GLUT-4), whereas MAPK is responsible for the mitogenic effect of insulin. Activated PI-3K triggers another signaling pathway by acting on phospholipids of the cell membrane to produce phosphatidylinositol 3,4,5-triphosphate. In turn, this molecule triggers a cascade of reactions involving protein kinase B (AKT) that are responsible for the translocation of GLUT-4 from the cytoplasm to the cell membrane, which facilitates diffusion of glucose into the cytoplasm and further transformation. Some glucose molecules undergo “rapid” intracellular metabolism and are utilized in the process of glycolysis and then cellular respiration. In contrast, the excess glucose is stored as a substrate for glycogenogenesis. On the other hand, activation of AKT leads to inhibition of protein kinase A (PKA) activity, and thus, inhibition of lipolysis and induction of lipogenesis, leading to triacylglycerol (TAG) storage in adipose tissue [6]. In the muscles, insulin increases amino acid uptake and enhances protein synthesis. In addition, it exhibits mitogenic properties, which promotes cell differentiation and growth. Insulin signaling also plays an important role in nitric oxide production, which is considered a strong vasodilator and a substance with anti-atherosclerosis properties [7].

### 2.2. IR Cell Classification

The molecular mechanisms of insulin described above provide the basis for understanding the pathogenesis of IR at the cellular level. Disturbance of signaling at any of these stages may lead to development IR, which can be classified as follows:

- Pre-receptor: genetically determined disorders associated with abnormal insulin structure or the presence of specific antibodies against insulin.
- Receptor: structural or functional defects in the insulin receptor (IRec).
- Post-receptor: impaired signaling after insulin binds to IRec characterized by impaired GLUT-4 translocation to the cell membrane and reduced transport of glucose into the cell interior.

The original hypothesis that proposed decreased IRec binding was responsible for the typical obesity-related IR has given way to a concept where defects in insulin signaling via IRec play a major role in the development of IR.

### 2.3. IR as a Disorder Secondary to Obesity

Obesity disease is the primary risk factor for IR, where the direct cause is an overload of adipose tissue with lipids, which translates into dysfunction. When adipose tissue loses its ability to store energy in the form of fat, it becomes a source of pro-inflammatory cytokines, dysregulating adipokine secretion and triggering the release of large amounts of free fatty acids (FFAs), which leads to the development of a chronic low-grade systemic inflammatory reaction in various tissues (adipose, muscle, liver and endothelium of blood vessels).

Subcutaneous adipose tissue (SAT) is made up of white adipose tissue where excess TAGs consumed with food are stored in a positive energy balance. TAG storage occurs by either increasing the volume of a single adipocyte (hypertrophic obesity) or recruiting new cells from precursor adipocytes (hyperplastic obesity). At this point, depending on individual sensitivity, peripheral obesity develops. However, when the storage capacity of the SAT is exceeded and recruitment of new adipocytes is no longer possible, TAGs also accumulate in areas outside SAT, such as visceral adipose tissue (VAT). As long as adipose tissue is capable of storing excess energy as a result of hypertrophy and/or hyperplasia, it is insulin sensitive, and overt metabolic disorders will not develop. However, when hypertrophic adipocytes lose the ability to store lipids, resistance to the antilipolytic effect of insulin develops. In this context, IR may be understood as a protective mechanism that is triggered in response to a significant increase in the accumulation of energy components. Currently, it seems that VAT is characterized by different features than SAT in terms of the type of tissue-building cells or metabolic properties that increases the risk of developing metabolic complications, led by IR, and a greater risk of cardiovascular disease [8,9,10].

**Figure 2:**
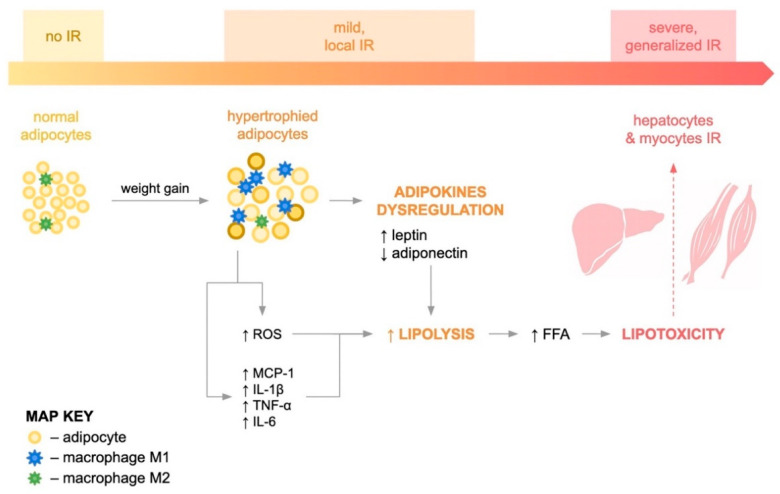
IR pathophysiology.

Hypertrophic adipocytes are responsible for adipose tissue ischemia and, consequently, its hypoxia, which leads to apoptosis of individual adipocytes and the gradual development of local inflammation, as shown in Figure 2. Another hypothesis is that the low-grade inflammation that accompanies obesity leads to impaired peripheral tissue insulin sensitivity. Increased plasma levels of inflammatory mediators, e.g., C-reactive protein, leukocytes or interleukin 6 (IL-6), have been shown to predict the development of insulin resistance and type 2 diabetes [11].

An important element of this inflammation seems to be the infiltration of adipose tissue by immune cells. Then, adipose tissue secretes several types of pro-inflammatory adipokines such as monocyte chemotactic protein-1 (MCP-1), tumor necrosis factor α (TNF-α), interleukin 1 beta (IL-1β) and interleukin 6 (IL-6). MCP-1 is a chemokine that is responsible for the migration of monocytes from blood to adipose tissue and their differentiation into proinflammatory macrophages (M1), and the transition of anti-inflammatory M2 macrophages into M1 macrophages in adipose tissue. This first stage induces local inflammation in adipose tissue and is responsible for the dysregulation of adipokine production, increasing the synthesis of proinflammatory substances (leptin, resistin) and reducing the synthesis of adiponectin, the only local insulin sensitizing and anti-inflammatory compound [12,13,14]. Studies have shown that, in humans, infiltration of adipose tissue by macrophages correlates with adipocyte size and BMI. In normal weight individuals, macrophages constitute approximately 10% of adipose tissue cells, whereas in obese individuals, the percentage of macrophages increases to 40–50%. Macrophage infiltration of adipose tissue in humans predominantly affects visceral adipose tissue rather than subcutaneous adipose tissue. This is supported by the fact that a low-calorie diet, exercise or surgical treatment reduces the percentage of adipose tissue macrophages in patients with morbid obesity [15,16,17].

Moreover, accumulation of lipids in adipocytes can induce oxidative stress, as evidenced by excess reactive oxygen species (ROS) such as hydrogen peroxide and hydroxyl radical ions by activating NADPH oxidase. Recent studies have shown that ROS-induced damage has lately been recognized as one of the key mechanisms in the pathogenesis of IR [13].

Dysfunctional adipose tissue is characterized by increased lipolytic activity, which results in the hydrolysis of TAGs and the release of FFAs into the bloodstream and antagonizes insulin-mediated metabolic processes. Therefore, the presence of circulating FFAs in the blood of obese people is a marker of adipose tissue insufficiency, proving that the capacity to store energy has been exceeded. Circulating FFAs and their metabolites, i.e., acetyl-CoA and diacylglycerols (DAGs), are taken up by peripheral cells such as myocytes and hepatocytes, where they accumulate ectopically. The effects of lipid accumulation in tissues other than adipose tissue are referred to as lipotoxicity and indicate systemic inflammation and IR [2,18]. It has been shown that DAGs activate protein kinase C (PKC), which allows the activity of serine/threonine kinases on IRec substrates, preventing tyrosine phosphorylation of IRS and altering post-receptor signaling [19]. Numerous studies have provided information on elevated ceramide concentrations in the liver and muscles of obese people compared to their healthy counterparts, which is probably due to increased intracellular availability of FFAs as substrates for ceramide synthesis. In vitro data indicate that ceramide inhibits insulin signaling, primarily through inactivation of protein kinase B, and in vivo data suggest that ceramide accumulation in muscle cells may be associated with the development of insulin resistance [20]. There is also a positive correlation between the total content of ceramides in SAT and the HOMA index. Interestingly, the study by Blachnio-Zabielska et al. found that inhibition of ceramide de novo production reverses IR, lowers the HOMA-IR value and improves glucose tolerance [21].

In addition to defective signaling, the development of IR is characterized by the dysfunction of mitochondria, the main organelles responsible for energy metabolism of individual cells. Boucher et al. showed increased mitochondrial fission in adipocytes when these cells were exposed to high levels of glucose or FFAs. Mitochondrial fission increases the expression of MAPK proteins and changes the activation of IRS-1 and Akt, which intensifies IR [22].

Such local pathologies eventually transform into a systemic process as peripheral tissues—mainly skeletal muscles and the liver—undergo similar inflammatory processes, which leads to the development of systemic IR.

## 3. Clinical Markers of IR

Isolated IR may be asymptomatic (subclinical), although most often it is revealed clinically as a symptom or a set of symptoms leading to the development of specific diseases. In clinical terms, it is very important to recognize IR as soon as possible and introduce specific prophylactic or therapeutic treatment methods.

The presence of obesity as measured by BMI and/or abdominal obesity as measured by waist circumference seems to be the first clinical symptom suggestive of IR in a patient. Therefore, several measurements should be performed according to Table 2. Visceral obesity, otherwise known as abdominal, android or central obesity, correlates with IR. Visceral obesity is characterized by the distribution of fat in the peritoneal cavity and between internal organs as opposed to subcutaneous fat in peripheral obesity.

In addition, the waist circumference and waist to height ratio (WHtR) can also be taken into account. The latter is a parameter assessing the cardiometabolic risk of obesity, and is more reliable than the waist circumference itself because it takes into account differences in height. Thus, it has recently become popular to say, “Keep your waist to less than half your height” [23].

A more advanced method for assessing body fat is a simple and non-invasive bioelectrical impedance analysis that defines obesity as a fat content of >25% in men and >35% in women, and overweight as >20% in men and >30% in women. On the other hand, the most accurate method is magnetic resonance imaging (diagnosis of obesity at a 100 cm^2^ visceral fat area), but this is mainly for research purposes.

Obese patients may also present with skin manifestations of insulin resistance that are possibly related to excess insulin-like growth factor type 1 (IGF-1). These include:

- acanthosis nigricans (pseudoacanthosis nigricans),
- keratosis pilaris (follicular keratosis),
- acrochordon (skin tags, soft fibromas),
- plantar hyperkeratosis.

**Figure 3:**
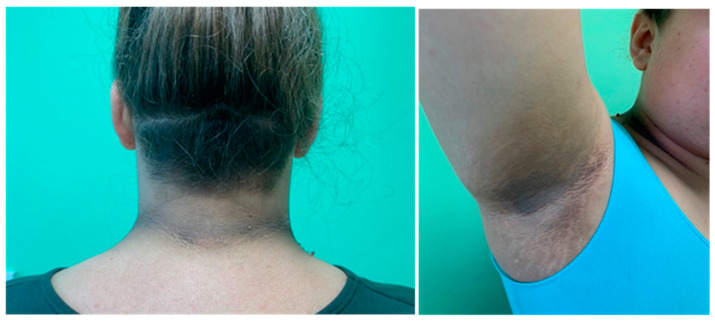
Acanthosis nigricans (authors’ data).

Acanthosis nigricans (AN) (Figure 3) is skin hyperpigmentation combined with excessive keratosis mainly located in the armpits, around the posterior neck, in the joint bends and in the umbilicus. The histopathological picture of AN has characteristic features where the differentiation concerns mainly inflammatory lesions with pruritus and superficial fungal infections, including intertrigo common in obese patients [24].

Keratosis pilaris (KP) is characterized by keratinous plugs in follicular orifices and varying degrees of perifollicular erythema. It is most commonly seen in the extensor surfaces of the upper arms, thighs and buttocks and is diagnosed during dermatoscopy. Though medically harmless, keratosis pilaris may be associated with certain genetic disorders [25].

Acrochordons are small, soft, usually pedunculated and benign skin tumors that are most often found on the neck, axilla or groin. They are protrusions of loose fibrous tissue and appear with increased frequency not only in patients with obesity or diabetes mellitus type 2, but also in acromegaly and colonic polyps [26].

Plantar hyperkeratosis presents as calluses, corns and different kinds of keratinized skin lesions, probably due to high pressure and inflammation. This skin disorder is more common in individuals aged over 60 years and should be considered a visible sign of severe obesity.

AN and KP seem to be reliable hyperinsulinemia markers in nondiabetic, obese patients [27].

In further clinical management, it is advisable to actively search for the features of metabolic syndrome due to the multitude of potential IR organ complications. According to IDF, metabolic syndrome is diagnosed when the patient suffers from central obesity (waist circumference of ≥94 cm in males, ≥80 cm in females) and at least two of the following abnormalities: hypertrigliceridemia (≥150 mg/dl), reduced HDL cholesterol (<40 mg/dl in males, <50 mg/dl in females), high blood pressure (systolic BP ≥ 130 or diastolic BP ≥ 85 mm Hg) and increased fasting plasma glucose (≥100 mg/dl). Treatment of these abnormalities is also included in the criteria [26].

IR is the main pathogenetic factor of carbohydrate disorders such as type 2 diabetes and pre-diabetes. The consequence of hepatic IR and no inhibition of gluconeogenesis is abnormal fasting glycemia (IFG), but when IR mainly affects skeletal muscles, then it is abnormal glucose tolerance (IGT). In the course of pre-diabetes (IFG, IGT), IR may be asymptomatic for a long time. However, the development of hyperglycemia, especially in combination with hyperlipidemia and chronic inflammation, leads to the initiation or acceleration of atherogenesis processes, development of atherosclerosis and consequently, a group of cardiovascular complications [28].

Other common obesity complications include hyperuricemia or gout, hypercoaguability, fatty liver, atherosclerosis, cardiovascular diseases (such as myocardial infarction and stroke), depression, sleep apnea, cancer and osteoarthritis [29].

In addition, IR is associated with the frequent occurrence of obesity in PCOS and complicates the course of this disease in patients with a normal body weight. Although IR is not included in the diagnostic criteria of PCOS, its markers often coexist and are associated with an increased risk of developing type 2 diabetes, dyslipidemia and atherosclerosis.

In men without type 2 diabetes, the relationship between IR and disorders of the reproductive system has not been established yet [3].

## 4. IR Laboratory Diagnostics

Despite frequent prevalence of IR in the population, no guidelines have been developed so far that would indicate a clear diagnosis of this pathology in the clinical setting. Therefore, in practice, it is necessary to combine information from the physical examination and the patient’s history with appropriately selected laboratory tests.

### 4.1. Hyperinsulinemic Euglycemic Glucose Clamp

According to many authors, the gold standard for the diagnosis of IR is a hyperinsulinemic euglycemic glucose clamp [30]. To perform this test, it is necessary to have access to two antecubital veins for infusing insulin and glucose and simultaneously collecting blood samples for determining glucose levels. Usually, the test lasts at least two hours during which constant hyperinsulinemia is achieved and gluconeogenesis in the liver is inhibited. The interpretation of the result takes into account the principle that the extent of IR is inversely proportional to glucose utilization. Due to the fact that said test is time-consuming, cost-intensive and must be performed under demanding conditions, it is hardly used in clinical practice [31].

### 4.2. Markers of Fasting Insulin

The first sign of laboratory-detectable metabolic disorders may be fasting hyperinsulinemia, which correlates well with IR, especially in normoglycemic patients [32]. It is worth emphasizing that only the fasting insulin concentration is relevant for the assessment of IR. The values obtained in the oral glucose tolerance test (OGTT) at 1 or 2 h have not been validated for the diagnosis of IR, and it seems that hyperinsulinemia after a glucose stimulus is a desirable metabolic effect. Despite this fact, some authors point to the potential advantage of the data obtained during the course of the OGTT [33].

However, it should be added that assessing insulinemia is difficult because insulin is secreted in a pulsatile manner, has a short half-life and analytical methods vary among laboratories. Moreover, in long-term type 2 diabetes, the reserves of pancreatic beta cells are depleted, hence the assessment of IR based on insulinemia in such patients may be unreliable [34].

### 4.3. HOMA

In clinical practice, a frequently used parameter of IR is the homeostatic model assessment (HOMA) developed by David Matthews et al. in 1985. It is a mathematical model that takes into account the concentration of fasting glucose and insulin. Although it correlates well with the results of the hyperinsulinemic euglycemic glucose clamp test, there are discrepancies in cut-offs and interpretation of the results. Thus, an isolated finding in the form of an abnormal HOMA-IR result may be the cause of IR overdiagnosis, especially in the absence of other metabolic disturbance components [35].

The disadvantage of HOMA is that it does not take into account peripheral and hepatic IR. To overcome this problem, a modification of the formula called HOMA2 [36] was developed. HOMA2 takes into account renal glucose losses and proinsulin concentration. It also assesses concentrations of C-peptides, which have a longer serum half-life compared to insulin [31,34].

In 2000, Katz et al. developed the quantitative insulin sensitivity check index (QUICKI). It provides an advantage for predicting IR in patients with type 2 diabetes and obesity but is unreliable in patients with type 1 diabetes.

In the diagnosis of IR, the McAuley index can also be used, which takes into account fasting insulinemia and TG concentration. According to the authors of a Korean study, the McAuley index best correlates with uncomplicated IR [37].

A relatively new parameter for assessing IR is the proinsulin to insulin ratio, which increases in the case of IR and type 2 diabetes. It proves the hyperactivity of secretory beta pancreatic cells, which slow down proinsulin processing. The majority of IR indices take into account fasting glucose and insulin concentrations; however, the dynamic values obtained during the oral glucose tolerance test (OGTT) are used for the Matsuda index developed in 1999 by Matsuda et al. This index estimates the ability of peripheral tissues, mainly muscle cells, to utilize large amounts of exogenous glucose [38,39,40].

In the absence of the validation of a single laboratory test, some authors advise performing several, preferably a combination of tests focused on central IR (e.g., HOMA2) and assessment of the secretory function of pancreatic beta cells [40].

### 4.4. Other Laboratory IR Markers

Due to the frequent coexistence of IR and atherogenic dyslipidemia, the TG/HDL cholesterol (TG/HDL-C) ratio can also be used.

Fasting laboratory assessment of insulin sensitivity (FLAIS) is characterized by high sensitivity and specificity for the detection of IR and shows a high correlation with the hyperinsulinemic euglycemic glucose clamp test. This method takes into account the number of erythrocytes and serum concentrations of alanine aminotransferase (ALT), C-peptide, sex hormone binding protein (SHBG), adiponectin and insulin-like growth factor-binding protein-1 (IGFBP-1). Although the clinical application of FLAIS is currently limited by access to some of the diagnostic tests it contains, it seems promising for the future.

Similar hope rests on the lipoprotein insulin resistance index (LPIR), a biomarker obtained on the basis of the plasma lipids. It shows a high correlation with the HOMA-IR index, glycated hemoglobin, arterial hypertension, BMI and dyslipidemia. Other advantages of this test include the wide availability of the test and no limitations related to the low repeatability of insulin concentration measurements [41].

Another laboratory test that correlates well with insulin resistance is the concentration of sex hormone binding globulin (SHBG). Decreased SHBG levels have been observed in patients with insulin resistance-associated diseases, including type 2 diabetes, MAFLD and PCOS [42].

### 4.5. IR Location Subtypes

The phenomenon of IR, besides cellular classification, can also be divided by location into central and peripheral. Since interventions that improve IR are organ specific (Table 3), it is vital to estimate which organs are resistant to insulin, as well as the level of insulin resistance in each organ.

Central IR is located in the liver. Hepatic insulin resistance refers to impaired sup-pression of glucose production by insulin in hepatocytes. This usually manifests as im-paired fasting glucose (IFG) and MAFLD. In contrast, peripheral IR reflects impaired glucose uptake by peripheral tissues—skeletal muscles, adipose tissue, among many others—that manifests as impaired glucose tolerance (IGT) and a tendency towards type 2 diabetes mellitus.

Although the hyperinsulinemic euglycemic glucose clamp is the gold standard for measuring whole-body insulin resistance, some laboratory markers can specify the location of IR. HOMA and QUICKI take into account fasting glucose and insulin concentrations to estimate hepatic (central) IR, whereas the Matsuda index focuses on the dynamic values obtained in oral glucose tolerance test (OGTT) to estimate the level of skeletal muscle (peripheral) IR [43].

Research shows that certain lifestyle modifications and pharmacotherapy choices can be more useful in organ-specific disorders. In the case of hepatic IR, metformin and a low-fat diet are preferred, and in case of skeletal muscle IR, physical activity and a Mediterranean diet have a better effect [44].

## 5. Treatment of IR

The literature addressing the problem of IR treatment is poor with most articles focusing on the treatment of components of metabolic syndrome. Given that IR can be treated as a symptom or consequence of obesity, the main causal treatment seems to be anti-obesity management, which consists of three main steps: lifestyle changes (nutritional treatment, exercise), pharmacotherapy and metabolic (bariatric) surgery.

### 5.1. Lifestyle Changes

The mainstay of treatment for overweight and obesity is to achieve a negative energy balance by changing eating habits and increasing physical activity. In nutritional treatment, it is recommended that:

- the diet should be low energy (low carbohydrate, low glycemic index, low fat),
- it should be individualized and dependent on the patient’s body weight and physical activity,
- and the meals should be regular and frequent (4–5 times a day).

Often there is a need to establish an individual plan and its further modifications by a dietician [45].

Regular physical activity with weight loss can reduce the impact of many cardiometabolic risk factors, such as hyperglycemia, IR, hypertension and dyslipidemia. The following activities are recommended:

- Aerobic exercise (30–60 min of moderate to high intensity most days of the week) to achieve weight and adipose tissue loss, including a reduction in visceral abdominal and ectopic fat around the heart and in the liver. Brisk walking, Nordic walking, swimming and cycling are recommended.
- Resistance training can promote a reduction in fat mass and an increase in muscle mass, i.e., lean body mass. In addition, it increases insulin sensitivity because the extra muscle mass requires greater utilization of glucose without the participation of insulin during exercise. Interestingly, the combination of aerobic and resistance training has been shown to translate into an almost 20% better effect than either regimen on its own [46,47].
- Increasing the intensity of exercise, including high intensity interval training (HIIT), may result in a greater increase in cardiorespiratory fitness and achieve similar benefits more quickly than moderate-intensity aerobic exercise.

The guidelines suggest long-term regular physical activity that is associated with either the maintenance of body weight or a modest reduction in body weight for people with overweight and obesity [48].

Although the updated recommendations include 30–60 min of physical activity several times a week, the goal is not just to achieve weight loss but to help reduce abdominal visceral fat and ectopic fat, increase muscle mass, increase cardiorespiratory fitness and improve cardiometabolic risk factors (not just cardiovascular morbidity risk in postmenopausal women).

In addition, regular physical activity can improve quality of life, mood disorders (e.g., depression, anxiety) as well as perception of the body image, although there is limited evidence for its effect on the improvement of mental health.

### 5.2. IR Pharmacotherapy

According to the Canadian Adult Obesity Clinical Practice guidelines, anti-obesity therapy should be considered for patients with either a BMI ≥ 30 kg/m^2^ or a BMI of ≥27 kg/m^2^ when associated with typical obesity complications [49]. Depending on the clinical picture and complications of obesity, an individually tailored first line anti-obesity drug is advised. The combination drug naltrexone/bupropion is recommended for patients with eating disorders, craving, depression or for smokers. However, in patients with concomitant diabetes, pre-diabetes, hypertension, obstructive sleep apnea or PCOS, the GLP-1 analogue liraglutide is recommended.

As noted earlier, in the presence of obesity with PCOS, the use of liraglutide is recommended. However, the treatment of IR in the context of PCOS for people with a normal body weight requires separate management. Although non-pharmacological treatment has a greater effect on reducing IR in female PCOS patients, off-label metformin is also used for this purpose [50]. The main effect of metformin in PCOS is to delay the development of pre-diabetes and diabetes, improve the lipid profile and lower blood pressure [51].

The literature recommends no pharmacological treatment for IR alone without obesity or other concomitant diseases.

There are no recommendations for the use of thiazolidinediones—drugs that improve insulin sensitivity—in female patients without type 2 diabetes.

The results of studies on specific combinations of bacteria in probiotic preparations have shown they can reduce IR [52].

## Figures and Tables

**Table 1:** Types and causes of [3].

Primary IR	Secondary IR
IR due to inappropriate lifestyle and diet (“hyper-FFA-emia”) Congenital forms of IR: Leprechaunism, Rabson–Mendenhall syndrome, type A IR syndrome, some lipodystrophies	Obesity Polycystic ovary syndrome (PCOS) States of excess counterregulatory hormones for insulin: stress, infections, pregnancy, Cushing’s syndrome, pheochromocytoma, acromegaly, glucagonoma Other endocrinopathies: prolactinoma, hypopituitarism, hyperthyroidism and hypothyroidism, primary hyperparathyroidism, primary hyperaldosteronism, congenital adrenal hyperplasia, hypogonadism (including Klinefelter syndrome and Turner syndrome) Drug-induced IR: glucocorticosteroids, antiretroviral drugs, oral contraceptives IR of immune etiology: anti-insulin antibodies, antibodies against insulin receptors in type B IR syndrome Miscellaneous: starvation, ketoacidosis, uremia, liver cirrhosis

**Table 2:** Anthropometric measurements for obesity assessment.

Measurement	Values for Recognition	Clinical Relevance
Height	—	—
Body mass	—	—
Body mass index (BMI)	Overweight: 25.0–29.9 kg/m^2^ Obese (Class I): 30.0–34.9 kg/m^2^ Obese (Class II): 35.0–39.9 kg/m^2^ Obese (Class III): ≥40.0 kg/m^2^	Diagnosing the degree of obesity or overweight
Waist circumference (WC)	≥94 cm in males ≥80 cm in females	Diagnosis of abdominal obesity and increased cardiometabolic risk
Waist to height ratio (WHtR)	≥0.5 in males and females	
Waist to hip ratio (WHR)	>0.9 in males >0.85 in females	

**Table 3:** Comparison of the two main IR subtypes.

Type of IR	Hepatic IR	Skeletal Muscle IR
Location	Central	Peripheral
Mechanism	Steatosis of hepatocytes → impaired inhibition of gluconeogenesis, increased glycogenolysis	Steatosis of myocytes → impaired glucose uptake by skeletal muscles
Typical complications	Impaired fasting glucose (IFG) MAFLD	Impaired glucose tolerance (IGT) Type 2 diabetes mellitus
Laboratory markers	HOMA, QUICKI	Matsuda index
Specific management	Metformin, low-fat diet	Physical activity, Mediterranean diet

## Data Availability

Not applicable.

## References

[1] Contreras P.H., Salgado A.M., Bernal Y.A., Vigil P. (2019). A Simple and Improved Predictor of Insulin Resistance Extracted From the Oral Glucose Tolerance Test: The I0*G60. J. Endocr. Soc..

[2] Yazıcı D., Sezer H. (2017). Insulin Resistance, Obesity and Lipotoxicity. Adv. Exp. Med. Biol..

[3] Koleva D.I., Orbetzova M.M., Atanassova P.K. (2013). Adipose tissue hormones and appetite and body weight regulators in insulin resistance. Folia Med. (Plovdiv).

[4] Gepstein V., Weiss R. (2019). Obesity as the Main Risk Factor for Metabolic Syndrome in Children. Front. Endocrinol..

[5] Sesti G. (2006). Pathophysiology of Insulin Resistance. Best Pract. Res. Clin. Endocrinol. Metab..

[6] Choi S.M., Tucker D.F., Gross D.N., Easton R.M., Dipilato L.M., Dean A.S., Monks B.R., Birnbaum M.J. (2010). Insulin Regulates Adipocyte Lipolysis via an Akt-Independent Signaling Pathway. Mol. Cell. Biol..

[7] Tokarz V.L., MacDonald P.E., Klip A. (2018). The Cell Biology of Systemic Insulin Function. J. Cell Biol..

[8] le Jemtel T.H., Samson R., Milligan G., Jaiswal A., Oparil S. (2018). Visceral Adipose Tissue Accumulation and Residual Cardiovascular Risk. Curr. Hypertens. Rep..

[9] Neeland I.J., Ross R., Després J.-P., Matsuzawa Y., Yamashita S., Shai I., Seidell J., Magni P., Santos R.D., Arsenault B. (2019). Review Visceral and Ectopic Fat, Atherosclerosis, and Cardiometabolic Disease: A Position Statement. Diabetes-Endocrinol..

[10] Ibrahim M.M. (2010). Subcutaneous and Visceral Adipose Tissue: Structural and Functional Differences. Obes. Rev..

[11] Shoelson S.E. (2006). Inflammation and Insulin Resistance. J. Clin. Investig..

[12] Kojta I., Chacińska M., Błachnio-Zabielska A. (2020). Obesity, Bioactive Lipids, and Adipose Tissue Inflammation in Insulin Resistance. Nutrients.

[13] Ahmed B., Sultana R., Greene M.W. (2021). Adipose Tissue and Insulin Resistance in Obese. Biomed. Pharmacother..

[14] Unamuno X., Gómez-Ambrosi J., Rodríguez A., Becerril S., Frühbeck G., Catalán V. (2018). Adipokine Dysregulation and Adipose Tissue Inflammation in Human Obesity. Eur. J. Clin. Investig..

[15] Harman-Boehm I., Blüher M., Redel H., Sion-Vardy N., Ovadia S., Avinoach E., Shai I., Klöting N., Stumvoll M., Bashan N. (2007). Macrophage Infiltration into Omental Versus Subcutaneous Fat across Different Populations: Effect of Regional Adiposity and the Comorbidities of Obesity. J. Clin. Endocrinol. Metab..

[16] Cancello R., Henegar C., Viguerie N., Taleb S., Poitou C., Rouault C., Coupaye M., Pelloux V., Hugol D., Bouillot J.-L. (2005). Reduction of Macrophage Infiltration and Chemoattractant Gene Expression Changes in White Adipose Tissue of Morbidly Obese Subjects After Surgery-Induced Weight Loss. Diabetes.

[17] Bruun J.M., Helge J.W., Richelsen B., Stallknecht B. (2006). Diet and Exercise Reduce Low-Grade Inflammation and Macrophage Infiltration in Adipose Tissue but Not in Skeletal Muscle in Severely Obese Subjects. Am. J. Physiol. -Endocrinol. Metab..

[18] Engin A.B., Engin A.B. (2017). What Is Lipotoxicity?. Adv. Exp. Med. Biol..

[19] Boden G. (2011). 45Obesity, Insulin Resistance and Free Fatty Acids. Curr. Opin. Endocrinol. Diabetes Obes..

[20] Straczkowski M., Kowalska I. (2008). The Role of Skeletal Muscle Sphingolipids in the Development of Insulin Resistance. Rev. Diabet. Stud..

[21] Blachnio-Zabielska A.U., Hady H.R., Markowski A.R., Kurianiuk A., Karwowska A., Górski J., Zabielski P. (2018). Inhibition of Ceramide De Novo Synthesis Affects Adipocytokine Secretion and Improves Systemic and Adipose Tissue Insulin Sensitivity. Int. J. Mol. Sci..

[22] Boucher J., Kleinridders A., Ronald Kahn C. (2014). Insulin Receptor Signaling in Normal and Insulin-Resistant States. Cold Spring Harb. Perspect. Biol..

[23] Ashwell M., Hsieh S.D. (2009). Six Reasons Why the Waist-to-Height Ratio Is a Rapid and Effective Global Indicator for Health Risks of Obesity and How Its Use Could Simplify the International Public Health Message on Obesity. Int. J. Food Sci. Nutr..

[24] Das A., Datta D., Kassir M., Wollina U., Galadari H., Lotti T., Jafferany M., Grabbe S., Goldust M. (2020). Acanthosis Nigricans: A Review. J. Cosmet. Dermatol..

[25] Thomas M., Khopkar U.S. (2012). Keratosis Pilaris Revisited: Is It More Than Just a Follicular Keratosis?. Int. J. Trichology.

[26] Consensus Statements. https://www.idf.org/e-library/consensus-statements/60-idfconsensus-worldwide-definitionof-the-metabolic-syndrome.html.

[27] Plascencia Gómez A., Vega Memije M.E., Torres Tamayo M., Rodríguez Carreón A.A. (2014). Skin Disorders in Overweight and Obese Patients and Their Relationship with Insulin. Actas Dermo-Sifiliográficas.

[28] Poznyak A., Grechko A.V., Poggio P., Myasoedova V.A., Alfieri V., Orekhov A.N. (2020). Molecular Sciences The Diabetes Mellitus-Atherosclerosis Connection: The Role of Lipid and Glucose Metabolism and Chronic Inflammation. Int. J. Mol. Sci..

[29] Ascaso J.F., Pardo S., Real J.T., Lorente R.I., Priego A., Carmena R. (2003). Diagnosing Insulin Resistance by Simple Quantitative Methods in Subjects with Normal Glucose Metabolism. Diabetes Care.

[30] Placzkowska S., Pawlik-Sobecka L., Kokot I., Piwowar A. (2019). Indirect Insulin Resistance Detection: Current Clinical Trends and Laboratory Limitations. Biomed. Pap. Med. Fac. Univ. Palacky. Olomouc Czech Repub.

[31] Muniyappa R., Lee S., Chen H., Quon M.J. (2008). Current Approaches for Assessing Insulin Sensitivity and Resistance in Vivo: Advantages, Limitations, and Appropriate Usage. Am. J. Physiol. Endocrinol. Metab..

[32] Mcauley K.A., Williams S.M., Mann J.I., Walker R.J., Lewis-Barned N.J., Temple L.A., Duncan A.W. (2001). Diagnosing Insulin Resistance in the General Population. Diabetes Care.

[33] Borai A., Livingstone C., Shafi S., Zarif H., Ferns G. (2010). Insulin Sensitivity (Si) Assessment in Lean and Overweight Subjects Using Two Different Protocols and Updated Software. Scand. J. Clin. Lab. Investig..

[34] Retnakaran R., Shen S., Hanley A.J., Vuksan V., Hamilton J.K., Zinman B. (2008). Hyperbolic Relationship Between Insulin Secretion and Sensitivity on Oral Glucose Tolerance Test. Obesity.

[35] Matthews D.R., Hosker J.P., Rudenski A.S., Naylor B.A., Treacher D.F., Turner R.C. (1985). Homeostasis Model Assessment: Insulin Resistance and β-Cell Function from Fasting Plasma Glucose and Insulin Concentrations in Man. Diabetologia.

[36] Levy J.C., Matthews D.R., Hermans M.P. (1998). Correct Homeostasis Model Assessment (HOMA) Evaluation Uses the Computer Program. Diabetes Care.

[37] Kim T.J., Kim H.J., Kim Y.B., Lee J.Y., Lee H.S., Hong J.H., Lee J.W. (2016). Comparison of Surrogate Markers as Measures of Uncomplicated Insulin Resistance in Korean Adults. Korean J. Fam. Med..

[38] Abdelsalam N.M. (2019). Proinsulin/Insulin Ratio as a Predictor of Insulin Resistance and B-Cell Dysfunction in Obese Egyptians ((Insulin Resistance & B-Cell Dysfunction in Obese Egyptians)). Diabetes Metab. Syndr. Clin. Res. Rev..

[39] Mezza T., Ferraro P.M., Sun V.A., Moffa S., Cefalo C.M.A., Quero G., Cinti F., Sorice G.P., Pontecorvi A., Folli F. (2018). Increased β-Cell Workload Modulates Proinsulin-to-Insulin Ratio in Humans. Diabetes.

[40] Płaczkowska S., Pawlik-Sobecka L., Kokot I., Piwowar A. (2020). Estimation of Reference Intervals of Insulin Resistance (HOMA), Insulin Sensitivity (Matsuda), and Insulin Secretion Sensitivity Indices (ISSI-2) in Polish Young People. Ann. Agric. Environ. Med..

[41] Chung S.T., Matta S.T., Meyers A.G., Cravalho C.K., Villalobos-Perez A., Dawson J.M., Sharma V.R., Sampson M.L., Otvos J.D., Magge S.N. (2021). Nuclear Magnetic Resonance Derived Biomarkers for Evaluating Cardiometabolic Risk in Youth and Young Adults Across the Spectrum of Glucose Tolerance. Front. Endocrinol..

[42] Wallace I.R., Mckinley M.C., Bell P.M., Hunter S.J. (2012). Sex Hormone Binding Globulin and Insulin Resistance. Clin. Endocrinol..

[43] Abdul-Ghani M.A., Matsuda M., Balas B., DeFronzo R.A. (2007). Muscle and Liver Insulin Resistance Indexes Derived From the Oral Glucose Tolerance Test. Diabetes Care.

[44] Blanco-Rojo R., Alcala-Diaz J.F., Wopereis S., Perez-Martinez P., Quintana-Navarro G.M., Marin C., Ordovas J.M., van Ommen B., Perez-Jimenez F., Delgado-Lista J. (2016). The Insulin Resistance Phenotype (Muscle or Liver) Interacts with the Type of Diet to Determine Changes in Disposition Index after 2 Years of Intervention: The CORDIOPREV-DIAB Randomised Clinical Trial. Diabetologia.

[45] Lindstrom J., Louheranta A., Mannelin M., Rastas M., Salminen V., Eriksson J., Finnish Diabetes Prevention Study Group (2003). The Finnish Diabetes Prevention Study (DPS) Lifestyle intervention and 3-year results on diet and physical activity. Diabetes Care.

[46] Kanaley J.A., Colberg S.R., Corcoran M.H., Malin S.K., Rodriguez N.R., Crespo C.J., Kirwan J.P., Zierath J.R. (2022). Exercise/Physical Activity in Individuals with Type 2 Diabetes: A Consensus Statement from the American College of Sports Medicine. Med. Sci. Sports Exerc..

[47] Paquin J., Lagacé J.C., Brochu M., Dionne I.J. (2021). Exercising for Insulin Sensitivity—Is There a Mechanistic Relationship with Quantitative Changes in Skeletal Muscle Mass?. Front. Physiol..

[48] Boulé N.G., Prud’homme D. Canadian Adult Obesity Clinical Practice Guidelines: Physical Activity in Obesity Management. https://obesitycanada.ca/guidelines/physicalactivity.

[49] Pedersen S.D., Manjoo P., Wharton S. Canadian Adult Obesity Clinical Practice Guidelines: Pharmacotherapy in Obesity Management. https://obesitycanada.ca/guidelines/pharmacotherapy.

[50] Tang T., Norman R.J., Balen A.H., Lord J.M. (2003). Insulin-Sensitising Drugs (Metformin, Troglitazone, Rosiglitazone, Pioglitazone, D-Chiro-Inositol) for Polycystic Ovary Syndrome. Cochrane Database Syst. Rev..

[51] Diabetes Prevention Program Research Group (2002). Reduction in the Incidence of Type 2 Diabetes with Lifestyle Intervention or Metformin. N. Engl. J. Med..

[52] Szulińska M., Łoniewski I., van Hemert S., Sobieska M., Bogdański P. (2018). Dose-Dependent Effects of Multispecies Probiotic Supplementation on the Lipopolysaccharide (LPS) Level and Cardiometabolic Profile in Obese Postmenopausal Women: A 12-Week Randomized Clinical Trial. Nutrients.

